# Predictors of long-term prognosis in acute kidney injury survivors who require continuous renal replacement therapy after cardiovascular surgery

**DOI:** 10.1371/journal.pone.0211429

**Published:** 2019-01-31

**Authors:** Keita Sueyoshi, Yusuke Watanabe, Tsutomu Inoue, Yoichi Ohno, Hiroyuki Nakajima, Hirokazu Okada

**Affiliations:** 1 Department of Nephrology, Saitama Medical University, Saitama, Japan; 2 Division of Dialysis Center and Department of Nephrology, Saitama Medical University International Medical Center, Saitama, Japan; 3 Musashiranzan Hospital, Saitama, Japan; 4 Department of Cardiovascular Surgery, Saitama Medical University, International Medical Center, Saitama, Japan; University of Sao Paulo Medical School, BRAZIL

## Abstract

The long-term prognosis of patients with postoperative acute kidney injury (AKI) requiring continuous renal replacement therapy (CRRT) after cardiovascular surgery is unclear. We aimed to investigate long-term renal outcomes and survival in these patients to determine the risk factors for negative outcomes. Long-term prognosis was examined in 144 hospital survivors. All patients were independent and on renal replacement therapy at hospital discharge. The median age at operation was 72.0 years, and the median pre-operative estimated glomerular filtration rate (eGFR) was 39.5 mL/min/1.73 m^2^. The median follow-up duration was 1075 days. The endpoints were death, chronic maintenance dialysis dependence, and a composite of death and chronic dialysis. Predictors for death and dialysis were evaluated using Fine and Gray’s competing risk analysis. The cumulative incidence of death was 34.9%, and the chronic dialysis rate was 13.3% during the observation period. In the multivariate proportional hazards analysis, eGFR <30 mL/min/1.73 m^2^ at discharge was associated with the composite endpoint of death and dialysis [hazard ratio (HR), 2.1; 95% confidence interval (CI), 1.1–3.8; *P* = 0.02]. Hypertension (HR 8.7, 95% CI, 2.2–35.4; *P* = 0.002) and eGFR <30 mL/min/1.73 m^2^ at discharge (HR 26.4, 95% CI, 2.6–267.1; *P* = 0.006) were associated with dialysis. Advanced age (≥75 years) was predictive of death. Patients with severe CRRT-requiring AKI after cardiovascular surgery have increased risks of chronic dialysis and death. Patients with eGFR <30 mL/min/1.73 m^2^ at discharge should be monitored especially carefully by nephrologists due to the risk of chronic dialysis and death.

## Introduction

Acute kidney injury (AKI) is a common and serious complication in hospitalized patients that increases short-term mortality [1, 2]. Patients with severe forms of AKI need acute dialysis, and the incidence of dialysis-requiring AKI (AKI-D) in the United States has increased rapidly in the past decades [3]. In recent years, several observational studies have reported that patients who survive after AKI are more likely to develop chronic kidney disease (CKD) and end-stage renal disease (ESRD), and have higher long-term mortality than patients without AKI [4–6]. Cardiovascular surgery is also associated with a high risk of postoperative AKI, which leads to poor short-term and long-term prognoses [7]. In the most severe AKI cases, continuous renal replacement therapy (CRRT) is performed if the patients’ hemodynamics are unstable. Cardiovascular surgery is a major cause of CRRT-requiring AKI [8]. However, reports of the long-term prognoses of patients who develop postoperative AKI and receive CRRT after cardiovascular surgery are scarce [9, 10]. Here, we report the long-term outcomes and renal prognoses of patients who developed postoperative AKI and required CRRT after cardiovascular surgery. Furthermore, we investigate the risk factors for death and chronic dialysis dependence using competing risks methods.

## Materials and methods

### Study population and data collection

A retrospective cohort study was performed at an academic hospital (Saitama Medical University International Medical Center, Japan). Patients who underwent cardiovascular surgery and developed severe AKI requiring CRRT between April 2007 and December 2014 were identified using records from the dialysis center. Patients who underwent renal replacement therapy (RRT) or kidney transplantation before admission were excluded. Individual medical records were reviewed and the following pre-operative clinical and demographic data were collected: age, sex, body weight, body mass index (BMI), serum creatinine (sCr), albumin, hemoglobin, Charlson comorbidity index (CCI) score, and presence or absence of diabetes mellitus and hypertension. The CCI score is a well validated method for estimating the risk of death from comorbid disease and has been used in previous multiple studies [11]. The variables that comprise the CCI score include myocardial infarction, congestive heart failure, peripheral vascular disease, and cerebrovascular disease. Each comorbid disease included in the CCI is assigned a score of 1, 2, 3, or 6 depending on the risk of death associated with each comorbid disease. The individual scores are summed to calculate a total comorbidity score for the prediction of mortality. Pre-operative sCr level was defined as the last recorded sCr value within 1 week before surgery. Estimated glomerular filtration rate (eGFR) was calculated using the formula for Japanese patients [12]: eGFR (mL/min/1.73 m^2^)  =  194×sCr^−1.094^×age^−0.287^ (×0.739 for females).

Peri-operative data included the need for emergency surgery, operation type (coronary artery bypass grafting [CABG], valve surgery, CABG combined with valve surgery, vascular surgery, and others), and the use and duration of cardio-pulmonary bypass.

AKI was diagnosed according to the Kidney Disease Improving Global Outcomes (KDIGO) criteria [13]. In brief, diagnosis was based on an increase in sCr of ≥0.3 mg/dL within 48 hours, an increase in sCr to ≥1.5-times the baseline known or presumed to have occurred within the prior 7 days, or urine volume <0.5 mL/kg/hour for 6 hours. In patients presenting with AKI, CRRT was initiated when the cardiovascular surgeon and nephrologist agreed that it was necessary for removing excess fluid or solute before the classical criteria for renal replacement therapy initiation start to appear (severe hyperkalemia, metabolic acidosis, pulmonary edema, or uremia). Intermittent hemodialysis was not performed for initial RRT in this group of patients because all were hemodynamically unstable. CRRT involved either continuous veno-venous hemodialysis, hemofiltration, or hemodiafiltration. Patients who recovered from AKI discontinued CRRT, and when recovery was not achieved and dialysis-dependent conditions persisted, they were shifted to chronic maintenance dialysis.

The patients' sCr levels and eGFR were measured at hospital discharge. The sCr levels at discharge and pre-operation were compared and recovery of kidney function was evaluated. No consensus on the definition of renal function recovery after AKI has been reached [14]. In this study, it was defined as a decline in sCr to pre-operative levels [15]. As a simple surrogate index for activities of daily living (ADL) and frailty at discharge, we determined whether patients could walk independently [16–18]. The study was approved by the medical ethical review board of the Saitama Medical University International Medical Center (reference number 15–235), which waived the requirement for informed consent because of the retrospective nature of the study. All procedures followed were in accordance with the ethical standards of the Helsinki Declaration of 1975, as revised in 2000.

### Study outcomes

We conducted a long-term prognostic analysis of patients who survived the surgery, were withdrawn from RRT by hospital discharge, and survived for at least 90 days after hospital discharge without re-initiation of RRT or re-hospitalization within 90 days to reduce bias related to the short-term prognosis and to analyze only those patients who have survived the acute phase [19].

The endpoints were: (1) a composite endpoint of death and chronic dialysis (death prior to initiation of chronic dialysis and initiation of chronic dialysis), (2) initiation of chronic dialysis, and (3) death (prior to initiation of dialysis). The outcome was censored if a patient had not reached the endpoints by the time of the last follow-up or was lost to follow-up. The patients were followed-up by telephone surveys with them or their families, or by correspondence with the hospital to which they were transferred. In cases where the prognosis could not be tracked fully during the observation period, it was censored at the last date on which the patient was known to be alive and had not started dialysis. In addition, we attempted to identify pre-, peri-, and postoperative factors related to long-term prognosis.

### Statistical analyses

The baseline characteristics of the study participants were analyzed using descriptive statistics. Continuous variables were expressed as the mean (standard deviation) or median (interquartile range [IQR]) and compared using the unpaired t-test or the Mann-Whitney U-test, respectively. The Shapiro-Wilk test was performed to test for normality of distribution. Categorical variables were expressed as percentages and compared using the chi-square test or Fisher’s exact test. The receiver operating characteristic (ROC) curve was constructed and the area under the curve (AUC) was calculated to determine the cut-off value of eGFR at hospital discharge for the prediction of chronic dialysis initiation, and age for the prediction of death. Curves for the composite of renal and patient survival were estimated for each category using Kaplan-Meier methods. The log-rank test was used to analyze differences between these curves with the Bonferroni post-hoc test. Cox regression analysis was used to evaluate independent predictors of composite long-term renal survival and patient survival. We analyzed for multicollinearity by assessing correlations between covariates with Spearman’s rank correlation coefficient.

### Competing risks methods

As death was a competing risk for the initiation of chronic dialysis, cumulative incidence curves were generated to estimate the risk of chronic dialysis and death using the Fine and Gray method, and a comparison was performed using Gray’s test [20–22]. Potential risk factors for increased long-term dialysis dependence and all-cause mortality were identified using univariate analysis. When variables were significant in univariate analysis (*P* <0.05) or deemed to be clinically important regardless of statistical significance, they were simultaneously included in the Fine-Gray’s proportional hazards model. Statistical analyses were performed using IBM SPSS software, version 24.0 (IBM Corp., Armonk, NY) and EZR software (Saitama Medical Center, Jichi Medical University, Saitama, Japan), which is a graphical user interface for R (version 3.0.2; The R Foundation for Statistical Computing, Vienna, Austria) [23]. More precisely, EZR is a modified version of R commander (version 2.0–3) that was designed to add statistical functions that are frequently used in biostatistics. A value of *P* <0.05 was considered to be significant.

## Results

### Clinical characteristics of study participants

Among 5,197 patients who underwent cardiovascular surgery between April 2007 and December 2014, 255 had severe AKI requiring CRRT. Ten patients (3.9%) died in the hospital and 28 (11.0%) were RRT-dependent at discharge. Among the remaining 217 hospital survivors, we identified 144 who survived to 90 days following hospital discharge with no re-initiation of dialysis and had a completely trackable clinical course. There were 6 patients who died within 90 days after discharge and 73 in which the prognosis could not be determined. As a result, the long-term follow-up rate was 68.2% (144/211 patients). The median age of patients at operation was 72.0 years (IQR: 64.0–79.0) and 73.6% were male. Median pre-operative sCr level was 1.37 mg/dL (IQR: 1.01–1.96) and median pre-operative eGFR was 39.5 mL/min/1.73 m^2^ (IQR: 24.3–51.9). Their demographic, comorbidity, and clinical characteristics are described in Table 1. Vascular operations, including endovascular procedures, were the most common type of surgery performed and included treatment for 26 cases of aortic dissection, 6 thoracic aortic aneurysms, 4 thoracoabdominal aortic aneurysms, and 7 abdominal aortic aneurysms.

**Table 1:** Baseline demographic, comorbidity, and pre-, peri-, and postoperative characteristics.

Variable			N = 144
**Demographics**			
	Age (years), median (IQR)	72.0 (64.0–79.0)	
	Male, n (%)	106 (73.6)	
	Body mass index (kg/m^2^), median (IQR)	24.0 (21.2–26.2)	
	Pre-op sCr (mg/dl), median (IQR)	1.37 (1.01–1.96)	
	Pre-op eGFR (ml/min/1.73 m^2^), median (IQR)	39.5 (24.3–51.9)	
	Pre-op CKD G3b–5, n (%)	89 (61.8)	
	Hb (g/dl)	11.5 ± 2.1	
	Alb (g/dl)	3.5 ± 0.6	
**Coexisting/** **previous** **conditions**			
	Diabetes mellitus, n (%)	73 (50.7)	
	Hypertension, n (%)	96 (66.7)	
	CCI, median (IQR)	2.0 (1.0–3.0)	
**Operation type**			
	Emergency op, n (%)	48 (33.3)	
	CPB, n (%)	78 (54.2)	
	CPB time (min), median (IQR)	178.0 (129.0–224.0)	
	Valve surgery, n (%)	24 (16.7)	
	CABG, n (%)	38 (26.4)	
	Valve+CABG, n (%)	12 (8.3)	
	Vascular surgery, n (%)	43 (29.9)	
	Others, n (%)	27 (18.8)	
**CRRT**			
	CRRT duration (days), median (IQR)	5.0 (3.0–8.0)	
**Condition at** **discharge**			
	sCr at discharge (mg/dl), median (IQR)	1.2 (0.91–1.65)	
	eGFR at discharge (ml/min/1.73 m^2^), median (IQR)	43.1 (26.0–61.6)	
	ADL at discharge (gait), n (%)	95 (66.0)	
	Recovery of kidney function at discharge, n (%)	78 (54.2)	

Continuous variables are shown as the mean ± SD or median (first to third quartile) and categorical variables as numbers (%). IQR, interquartile range; sCr, serum creatinine; eGFR, estimated glomerular filtration rate; CKD, chronic kidney disease; Hb, hemoglobin; Alb, albumin; CCI, Charlson comorbidity index; CPB, cardiopulmonary bypass; CABG, coronary artery bypass grafting; CRRT, continuous renal replacement therapy; ADL, activities of daily living.

The median follow-up time of the hospital survivors was 1075 days after discharge (IQR: 621–1,754; minimum: 91; maximum: 2,930). The last event occurred on day 2251, after which no deaths or dialysis initiations occurred. Among the 144 survivors, 9.0% (13 patients) had dialysis-dependent ESRD and 25.0% (36 patients) died during the observation period. Patients who progressed to chronic dialysis had significantly higher pre-operative sCr levels, lower pre-operative eGFR, and a higher ratio of CKD Grade 3b–5 (100% *vs*. 58.0%, *P* <0.001), and were more likely to have hypertension (92.3% *vs*. 64.1% *P* = 0.04), higher sCr levels, and lower eGFR at discharge (Table 2). Patients who died during follow-up were significantly older (76.0 *vs*. 70.0 years, *P* = 0.009) and had lower pre-operative BMI (22.2 *vs*. 24.7 kg/m^2^, *P* = 0.003) (Table 3).

**Table 2:** Baseline characteristics of the cohort stratified by progression to chronic dialysis.

Variable		Chronic dialysis N = 13	No chronic dialysis N = 131	*P* value
**Demographics**				
	Age (years), median (IQR)	72.0 (63.5–76.0)	73.0 (64.0–79.0)	0.4
	Male, n (%)	12 (92.3)	94 (71.8)	0.1
	Body mass index (kg/m^2^), median (IQR)	25.4 (22.0–27.3)	24.0 (21.1–26.1)	0.4
	Pre-op sCr (mg/dl), median (IQR)	2.58 (2.36–3.08)	1.31 (0.98–1.77)	<0.001*
	Pre-op eGFR (ml/min/1.73 m^2^), median (IQR)	19.4 (16.3–21.7)	41.2 (26.0–53.5)	<0.001*
	Pre-op CKD G3b–5, n (%)	13 (100)	76 (58.0)	0.003*
	Hb (g/dl)	11.6 ± 2.0	11.5 ± 2.1	0.9
	Alb (g/dl)	3.5 ± 0.4	3.5± 0.6	0.9
**Coexisting/previous** **conditions**				
	Diabetes mellitus, n (%)	9 (69.2)	64 (48.9)	0.2
	Hypertension, n (%)	12 (92.3)	84 (64.1)	0.04*
	CCI, median (IQR)	3.0 (2.0–4.0)	2.0 (1.0–3.0)	0.1
**Operation type**				
	Emergency op, n (%)	6 (46.2)	42 (32.1)	0.3
	CPB, n (%)	5 (38.5)	73 (55.7)	0.2
	CPB time (min), median (IQR)	142.0 (101.5–252.5)	178.0 (129.8–221.8)	0.8
	Valve surgery, n (%)	1 (7.7)	23 (17.6)	NA
	CABG, n (%)	5 (38.5)	33 (25.2)	NA
	Valve+CABG, n (%)	0 (0)	12 (9.2)	NA
	Vascular surgery, n (%)	6 (46.2)	37 (28.2)	NA
	Others, n (%)	1 (7.7)	26 (19.8)	NA
**CRRT**				
	CRRT duration (days), median (IQR)	6.0 (3.5–9.5)	5.0 (3.0–8.0)	0.5
**Condition at discharge**				
	sCr at discharge (mg/dl), median (IQR)	3.28 (2.38–4.46)	1.11 (0.87–1.59)	<0.001*
	eGFR at discharge (ml/min/1.73 m^2^), median (IQR)	15.8 (11.3–20.8)	46.5 (31.4–65.1)	<0.001*
	ADL at discharge (gait), n (%)	10 (76.9)	85 (64.9)	0.4
	Recovery of kidney function at discharge, n (%)	4 (30.8)	74 (56.5)	0.1

*P <0.05

IQR, interquartile range; sCr, serum creatinine; eGFR, estimated glomerular filtration rate; CKD, chronic kidney disease; Hb, hemoglobin; Alb, albumin; CCI, Charlson comorbidity index; CPB, cardiopulmonary bypass; CABG, coronary artery bypass grafting; CRRT, continuous renal replacement therapy; ADL, activities of daily living; NA, not analyzed

**Table 3:** Baseline characteristics of the cohort stratified by death during the follow-up period.

Variable		Death N = 36	Alive N = 108	*P* value
**Demographics**				
	Age (years), median (IQR)	76.0 (69.5–81.0)	70.0 (63.0–77.8)	0.009*
	Male, n (%)	25 (69.4)	81 (75.0)	0.5
	Body mass index (kg/m^2^), median (IQR)	22.2 (20.7–24.3)	24.7 (21.6–26.8)	0.003*
	Pre-op sCr (mg/dl), median (IQR)	1.37 (0.97–2.01)	1.36 (1.04–1.94)	0.9
	Pre-op eGFR (ml/min/1.73 m^2^), median (IQR)	39.5 (24.0–54.6)	39.8 (24.3–51.0)	0.9
	Pre-op CKD G3b–5, n (%)	22 (61.1)	67 (62.0)	0.9
	Hb (g/dl)	11.0 ±1.6	11.7 ± 2.2	0.2
	Alb (g/dl)	3.4 ± 0.5	3.5 ± 0.6	0.1
**Coexisting/previous conditions**				
	Diabetes mellitus, n (%)	18 (50.0)	55 (50.9)	0.9
	Hypertension, n (%)	23 (63.9)	73 (67.6)	0.7
	CCI, median (IQR)	2.0 (1.0–4.0)	2.0 (1.0–3.0)	0.2
**Operation type**				
	Emergency op, n (%)	12 (33.3)	36 (33.3)	1.0
	CPB, n (%)	21 (58.3)	57 (52.8)	0.6
	CPB time (min), median (IQR)	174.0 (150.0–202.0)	178.0 (124.0–236.0)	0.9
	Valve surgery, n (%)	4 (11.1)	20 (18.5)	NA
	CABG, n (%)	4 (11.1)	34 (31.5)	NA
	Valve+CABG, n (%)	5 (13.9)	7 (6.5)	NA
	Vascular surgery, n (%)	12 (33.3)	31 (28.7)	NA
	Others, n (%)	11 (30.6)	16 (14.8)	NA
**CRRT**				
	CRRT duration (days), median (IQR)	5.5 (3.3–10.5)	5.0 (3.0–7.0)	0.5
**Condition at discharge**				
	sCr at discharge (mg/dl), median (IQR)	1.35 (0.91–2.11)	1.15 (0.89–1.63)	0.3
	eGFR at discharge (ml/min/1.73 m^2^), median (IQR)	39.1 (22.9–55.0)	43.3 (26.8–64.8)	0.2
	ADL at discharge (gait), n (%)	21 (58.3)	74 (68.5)	0.3
	Recovery of kidney function at discharge, n (%)	15 (41.7)	63 (58.3)	0.1

*P <0.05

IQR, interquartile range; sCr, serum creatinine; eGFR, estimated glomerular filtration rate; CKD, chronic kidney disease; Hb, hemoglobin; Alb, albumin; CCI, Charlson comorbidity index; CPB, cardiopulmonary bypass; CABG, coronary artery bypass grafting; CRRT, continuous renal replacement therapy; ADL, activities of daily living; NA, not analyzed

The ROC curve identified the cut-off values of eGFR at discharge to be 24.9 ml/min/1.73 m^2^ (AUC = 0.93, sensitivity = 92.3%, specificity = 84.7%) for the prediction of chronic dialysis and 31.9 ml/min/1.73 m^2^ (AUC = 0.68; sensitivity = 54.3%; specificity = 76.5%) for the prediction of the composite of chronic dialysis and death. Based on the cut-off values in a previous report [10], in addition to our data, we set an eGFR of 30 ml/min/1.73 m^2^ at discharge as the cut-off value for risk stratification. Similarly, the ROC curve identified the cut-off value of age for the prediction of death to be 75.0 years (AUC = 0.64; sensitivity = 60.6%; specificity = 63.0%). Therefore, we used that cut-off value for stratification. Table 4 shows the patients’ demographic, comorbidity, and clinical parameters according to whether their eGFR was <30 ml/min/1.73 m^2^ or ≥30 ml/min/1.73 m^2^ at hospital discharge. Patients with eGFR <30 ml/min/1.73 m^2^ had lower preoperative serum albumin, a lower recovery from AKI rate, and more frequently had diabetes.

**Table 4:** Baseline characteristics of the cohort stratified by eGFR at hospital discharge.

Variable		eGFR <30 N = 41	eGFR ≥30 N = 103	*P* value
**Demographics**				
	Age (years), median (IQR)	72.0 (67.0–78.0)	72.0 (63.0–79.0)	0.9
	Male, n (%)	26 (63.4)	80 (77.7)	0.1
	Body mass index (kg/m^2^), median (IQR)	23.6 (21.5–26.0)	24.2 (21.0–26.2)	0.7
	Pre-op sCr (mg/dl), median (IQR)	2.12 (1.75–2.47)	1.23 (0.95–1.47)	<0.001*
	Pre-op eGFR (ml/min/1.73 m^2^), median (IQR)	22.4 (19.3–25.9)	45.5 (35.0–54.8)	<0.001*
	Pre-op CKD G3b–5, n (%)	38 (92.7)	51 (49.5)	<0.001*
	Hb (g/dl)	11.0 ±1.7	11.5 ± 2.2	0.1
	Alb (g/dl)	3.3 ± 0.5	3.6 ± 0.6	0.04*
**Coexisting / previous conditions**				
	Diabetes mellitus, n (%)	28 (68.3)	45 (43.7)	0.008*
	Hypertension, n (%)	32 (78.0)	64 (62.1)	0.1
	CCI, median (IQR)	2.0 (1.0–4.0)	2.0 (1.0–3.0)	0.1
**Operation type**				
	Emergency op, n (%)	13 (31.7)	35 (34.0)	0.8
	CPB, n (%)	17 (41.5)	61 (59.2)	0.1
	CPB time (min), median (IQR)	142.0 (96.0–209.0)	183.5 (135.8–231.5)	0.2
	Valve surgery, n (%)	8 (19.5)	16 (15.5)	NA
	CABG, n (%)	15 (36.6)	23 (22.3)	NA
	Valve+CABG, n (%)	1 (2.4)	11 (10.7)	NA
	Vascular surgery, n (%)	9 (22.0)	34 (33.0)	NA
	Others, n (%)	8 (19.5)	19 (18.4)	NA
**CRRT**				
	CRRT duration (days), median (IQR)	5.0 (3.8–9.3)	5.0 (3.0–7.3)	0.5
**Condition at discharge**				
	sCr at discharge (mg/dl), median (IQR)	2.4 (1.75–3.10)	1.03 (0.79–1.32)	<0.001*
	eGFR at discharge (ml/min/1.73 m^2^), median (IQR)	20.9 (16.5–24.3)	53.6 (39.5–69.2)	<0.001*
	ADL at discharge (gait), n (%)	10 (24.4)	39 (37.9)	0.1
	Recovery of kidney function at discharge, n (%)	12 (29.3)	66 (64.1)	<0.001*

*P <0.05

IQR, interquartile range; sCr, serum creatinine; eGFR, estimated glomerular filtration rate; CKD, chronic kidney disease; Hb, hemoglobin; Alb, albumin; CCI, Charlson comorbidity index; CPB, cardiopulmonary bypass; CABG, coronary artery bypass grafting; CRRT, continuous renal replacement therapy; ADL, activities of daily living; NA, not analyzed

### Outcomes

#### Cumulative incidences of the composite of death and initiation of chronic dialysis

**Fig 1:**
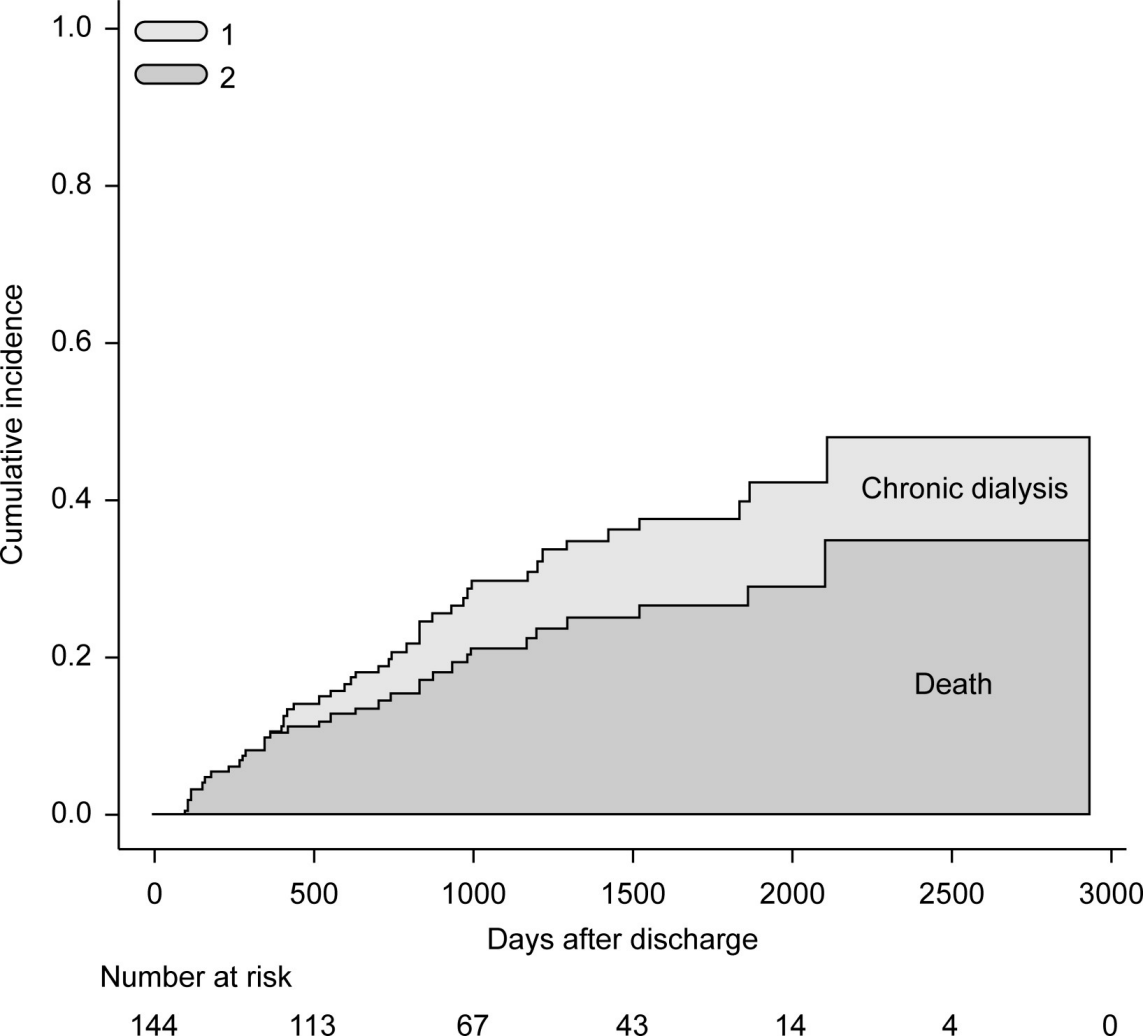
Cumulative incidences of the composite of death and initiation of chronic dialysis. (1) Indicates the cumulative incidence of initiation of chronic dialysis (13.3%) and (2) indicates the cumulative incidence of death (34.9%) during the observation period. The cumulative incidence of the composite endpoint of death and initiation of chronic dialysis was 48.2%.

Fig 1 shows the cumulative incidences of death and initiation of chronic dialysis evaluated using Gray's method, considering each as a competing risk. The cumulative incidence of death was 34.9% and that of chronic dialysis was 13.3% during the observation period. The cumulative incidence of the composite of death and initiation of chronic dialysis was 48.2%.

#### Predictors of the composite endpoint of death and initiation of dialysis

**Fig 2:**
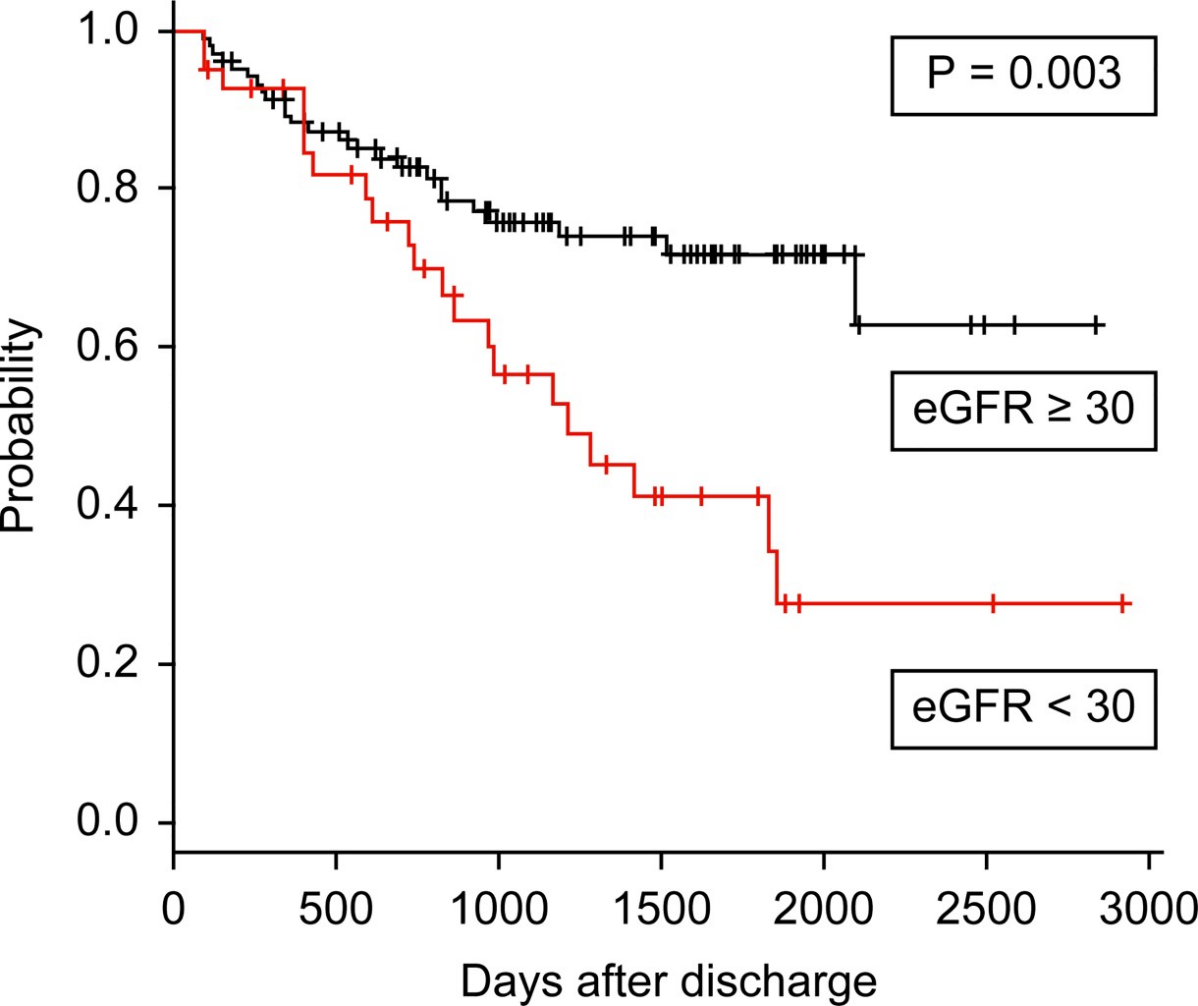
Kaplan-Meier survival curves depicting the period between discharge and the composite endpoint of death and chronic dialysis initiation. Association between estimated glomerular filtration rate (eGFR) ≥ or <30 mL/min/1.73 m^2^ at hospital discharge and composite endpoint.

Fig 2 shows the Kaplan-Meier survival curves depicting the period between discharge and the composite endpoint of death and chronic dialysis initiation. Compared with patients discharged with an eGFR ≥30 mL/min/1.73 m^2^, the long-term composite of life and renal survival was worse in patients with eGFR <30 mL/min/1.73 m^2^ at discharge in a log-rank test (*P* = 0.003) (Fig 2). Similarly, patients discharged with recovered kidney function after AKI had greater long-term composite life and renal survival, compared to patients with no recovery of kidney function (*P* = 0.02).

Univariate analysis identified that eGFR <30 mL/min/1.73 m^2^ and no recovery of renal function after AKI at discharge were significantly associated with the composite endpoint of death and initiation of dialysis. After multivariate Cox proportional hazards analysis, only eGFR <30 mL/min/1.73 m^2^ at discharge was associated with the composite endpoint of death and initiation of dialysis (hazard ratio, 2.1; 95% CI, 1.1–3.8; *P* = 0.02) (Table 5).

**Table 5:** Univariate and multivariate analysis of variables associated with a composite endpoint of death and initiation of chronic dialysis.

Variables		Univariate Analysis			Multivariate Analysis		
		HR (95% CI)		*P* value	HR (95% CI)		*P* value
**eGFR at discharge**	<30/≥30	2.4	(1.3–4.2)	0.005*	2.1	(1.1–3.8)	0.02*
**CCI**	≥3/<3	1.6	(0.9–2.9)	0.1	1.9	(1.0–3.7)	0.06
**Recovery of kidney function**	(–/+)	1.9	(1.1–3.5)	0.03*	1.8	(1.0–3.4)	0.06
**Age**	≥75/<75	1.6	(0.9–2.8)	0.1	1.6	(0.8–3.0)	0.2
**ADL at discharge (gait)**	(–/+)	1.3	(0.7–2.4)	0.4	1.5	(0.8–2.8)	0.2
**BMI**	<24/≥24	1.4	(0.8–2.5)	0.3	1.3	(0.7–2.5)	0.4
**Diabetes mellitus**	(+/–)	1.2	(0.7–2.2)	0.5	0.8	(0.4–1.6)	0.5
**Hypertension**	(+/–)	1.3	(0.7–2.5)	0.4	1.0	(0.5–2.0)	0.9

*P <0.05

HR, hazard ratio; CI, confidence interval; eGFR, estimated glomerular filtration rate; CCI, Charlson comorbidity index; ADL, activities of daily living; BMI, body mass index

#### Predictors of initiation of chronic dialysis

**Fig 3:**
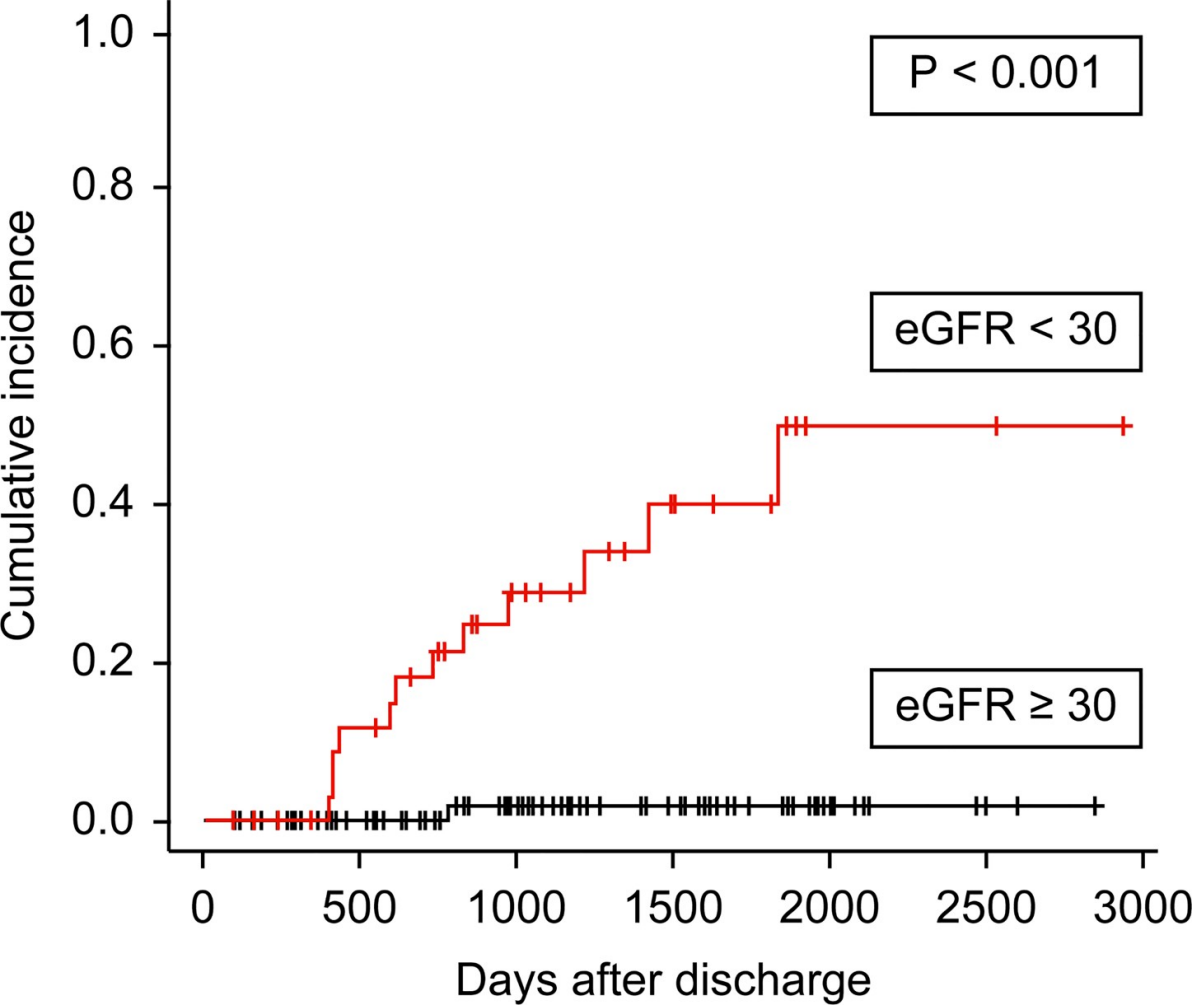
Cumulative incidences curves depicting period of time between discharge and chronic dialysis initiation censored for death. Association between estimated glomerular filtration rate (eGFR) ≥30 or <30 mL/min/1.73 m^2^ at hospital discharge and chronic dialysis initiation.

Fig 3 shows cumulative incidence curves depicting the period between discharge and chronic dialysis initiation, censored for death. Compared with patients discharged with an eGFR ≥30 mL/min/1.73 m^2^, long-term renal survival in patients with an eGFR <30 mL/min/1.73 m^2^ at discharge was worse in a Gray’s test (*P* <0.001) (Fig 3). Patients without hypertension had greater long-term renal survival (*P* = 0.02). Similarly, compared to patients with a pre-operative CCI <3, renal survival in patients with CCI ≥3 was worse (*P* = 0.03). Patients discharged with recovered kidney function after AKI had greater long-term renal survival compared to patients with no recovery of kidney function (*P* = 0.04).

In multivariable analysis using a Fine and Gray regression model that treated death as a competing event, hypertension (hazard ratio, 8.7; 95% CI, 2.2–35.4; *P* = 0.002) and eGFR <30 mL/min/1.73 m^2^ at discharge (hazard ratio, 26.4; 95% CI, 2.6–267.1, *P* = 0.006) were significantly associated with decreased renal survival (Table 6).

**Table 6:** Multivariate analyses of variables associated with initiation of chronic dialysis and death.

Variables		Chronic dialysis			Death		
		HR (95% CI)		*P* value	HR (95% CI)		*P* value
**eGFR at discharge**	<30/≥30	26.4	(2.6–267.1)	0.006*	0.9	(0.4–1.9)	0.7
**CCI**	≥3/<3	3.5	(0.4–30.4)	0.3	1.7	(0.7–3.6)	0.2
**Recovery of kidney function**	(–/+)	2.3	(0.5–10.5)	0.3	1.8	(0.9–3.5)	0.1
**Age**	≥75/<75	0.5	(0.13–2.2)	0.4	2.1	(1.0–4.6)	0.04*
**ADL at discharge (gait)**	(–/+)	0.5	(0.1–2.3)	0.4	1.7	(0.8–3.5)	0.2
**BMI**	<24/≥24	1.3	(0.3–5.3)	0.8	1.6	(0.8–3.4)	0.2
**Diabetes mellitus**	(+/–)	0.8	(0.2–4.2)	0.4	0.7	(0.3–1.5)	0.4
**Hypertension**	(+/–)	8.7	(2.2–35.4)	0.002*	0.7	(0.3–1.5)	0.4

*P <0.05

HR, hazard ratio; CI, confidence interval; eGFR, estimated glomerular filtration rate; CCI, Charlson comorbidity index; ADL, activities of daily living; BMI, body mass index

#### Predictors of death

**Fig 4:**
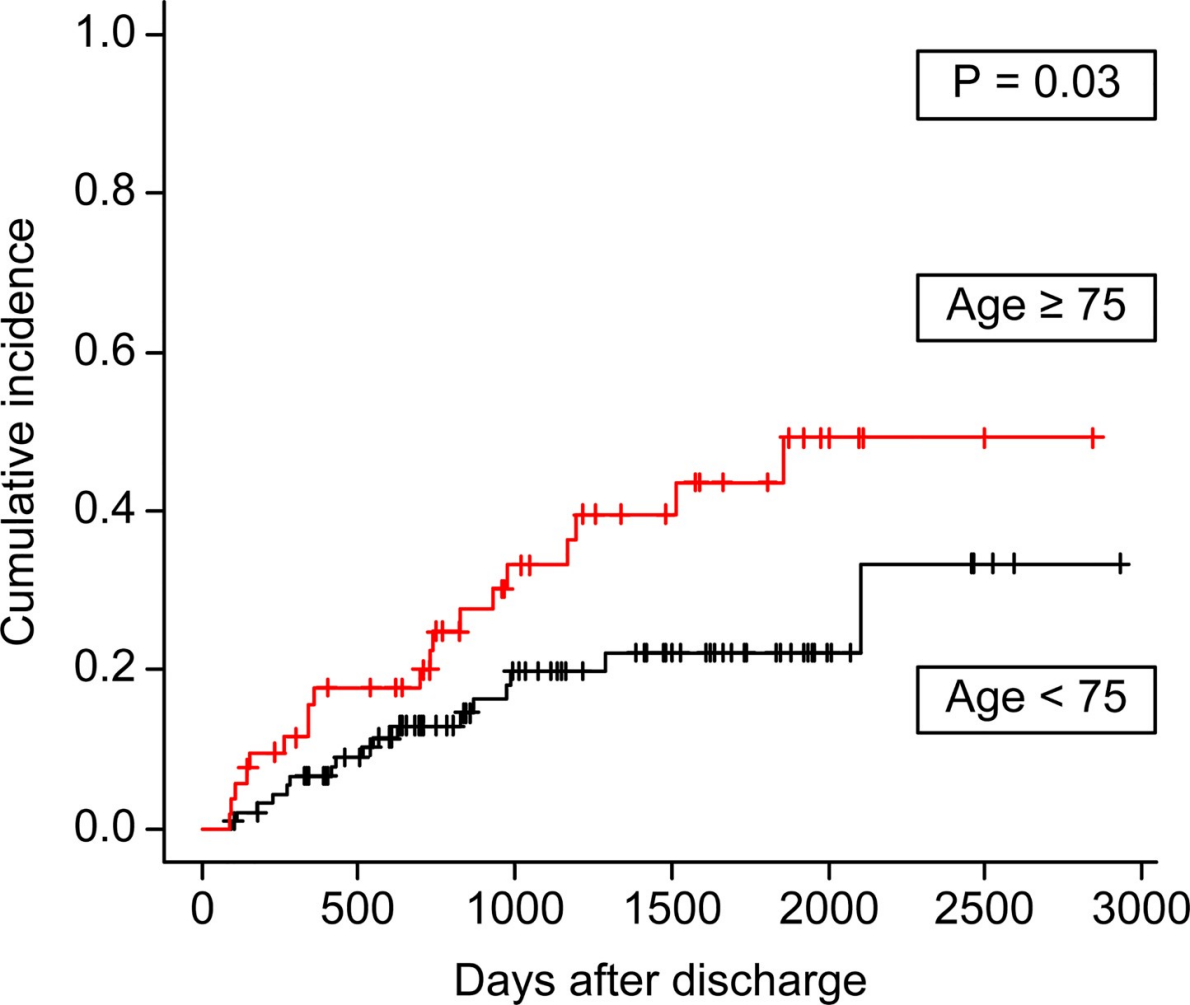
Cumulative incidence curves for death before initiation of chronic dialysis. Association between age ≥ or <75 years and death.

Fig 4 shows the cumulative incidence curves depicting the period between hospital discharge and death. Compared with patients aged <75 years, those aged ≥75 years had worse survival (*P* = 0.03) (Fig 4). The survival curves for patients incapable of walking (poor ADL) at hospital discharge tended to be worse, but this was not significant on Gray’s test (*P* = 0.2). Compared to patients with BMI ≥24 kg/m^2^, those with BMI <24 kg/m^2^ had worse survival (*P* = 0.04). When divided into four groups according to BMI (underweight, ≤18.5 kg/m^2^; normal, >18.5 to ≤25 kg/m^2^; overweight, >25 to ≤30 kg/m^2^; and obese, >30 kg/m^2^), long-term survival was significantly worse in the underweight group and better in the overweight group (*P* <0.001).

Fine and Gray’s multivariate proportional hazards analysis showed that only age ≥75 was significantly associated with worse long-term survival (hazard ratio, 2.1; 95% CI, 1.0–4.6; *P* = 0.04) (Table 6).

## Discussion

This study aimed to investigate the long-term survival and renal prognosis in patients with postoperative AKI requiring CRRT after cardiovascular surgery and to determine the risk factors for these outcomes. We found that survivors of severe AKI remained at a high risk of death and CKD progression. The cumulative incidence of death was 34.9% and that of chronic dialysis was 13.3% during the observation period. In univariate analysis, eGFR <30 mL/min/1.73 m^2^ and no recovery of renal function after AKI at discharge were significantly associated with the composite endpoint of death and initiation of dialysis. After multivariate Cox proportional hazards analysis, only eGFR <30 mL/min/1.73 m^2^ at discharge was associated with the composite endpoint. An eGFR <30 mL/min/1.73 m^2^ at discharge combined with hypertension was a strong predictor of chronic dialysis. Furthermore, advanced age (≥75 years) was also predictive of death. These data can be used to identify patients at a high risk of death and progressive CKD who may benefit from closer follow-up by nephrologists after hospital discharge.

A recently published systematic review and meta-analysis showed that patients who survive AKI have a greater risk of CKD, ESRD, and death compared to patients without AKI [14]. The risk of CKD or ESRD increased with the severity of AKI, advanced age, diabetes mellitus, hypertension, heart failure, increased CCI during AKI episodes, and low serum albumin levels [6, 24]. The presence of CKD before AKI and lack of recovery of kidney function after AKI are also associated with long-term renal and survival outcomes [14, 25]. The incidence of AKI-D has increased rapidly in the past decade; from 2000 to 2009, the incidence in the United States has increased at a yearly rate of 10% [3]. It is expected that the number of cases of AKI-D will continue to rise [26]. In other words, it is believed that the number of AKI-D patients who survive AKI and discharge will increase, making it difficult for nephrologists to cope with the increased demand for care. Therefore, identifying AKI-D survivors at risk of progressive CKD will help guide therapeutic intervention [19, 27].

Cardiac surgery is associated with a high risk for the development of AKI [28]. A recent systematic review and meta-analysis of 91 observational studies showed that the pooled incidence rate of AKI was 22.3% and that 2.3% of patients received acute RRT. The pooled AKI-associated long-term mortality rate was as high as 30% and increased with AKI severity [7]. In that study, the average patient age increased depending on the AKI diagnostic criteria used (RIFLE < AKIN < KDIGO). This suggests that in recent years, cardiac surgery is being more frequently performed in older patients, with the emergence of older AKI-D patients as a result. Few previous studies have investigated the long-term outcomes after RRT for cardiac surgery-associated AKI [9, 10]. Stads et al. [10] evaluated the association between impaired kidney function at hospital discharge and long-term renal survival and overall patients survival in AKI patients who received CRRT (25% of the cohort were post-thoracic surgery patients). They found that eGFR <30 mL/min/1.73 m^2^ at hospital discharge was a strong predictor for decreased long-term patients survival and poor renal survival [10]. Furthermore, Harel et al. [19] analyzed 4,383 survivors of dialysis-requiring AKI, some of whom underwent cardiac and aortic surgeries. In those who initially became dialysis-independent, the subsequent need for chronic maintenance dialysis was predicted by pre-existing CKD, hypertension, and global comorbidities evaluated by the CCI.

We believe that this study has several strengths. We analyzed the prognosis of elderly post-cardiac surgery patients, a population that is expected to increase in the future. We believe that the risks of chronic dialysis and death are more accurately evaluated with competing risk models [19, 20]. Most of our results are consistent with those from previous reports, with a few exceptions [10, 14]. As Stads et al had reported [10], impaired kidney function at hospital discharge was associated with not only long-term renal prognosis but also a composite renal and overall survival in our analyses. In addition, we found that hypertension was a strong predictor of CKD progression, as previously described [24]. Patients with hypertension before cardiac surgery may potentially have hypertensive renal impairment, even if their preoperative sCr and eGFR are within the normal range. It seems that the simultaneous occurrence of AKI accelerated the decline in kidney function [29]. On the other hand, there was no significant association between preoperative diabetes mellitus and long-term prognosis. This finding may have been influenced by the lack of data on the duration of diabetes mellitus and blood glucose control. Diabetic patients also have a high frequency of re-hospitalization, early death, and early dialysis within 90 days of discharge. Furthermore, in our study, older age was associated with poor long-term survival but not long-term renal prognosis. Most of the patients analyzed in this study were elderly (65 years or older), and it is possible that several had died before initiation of chronic dialysis. Finally, we believe that setting the eGFR cutoff value to 30 mL/min/1.73 m^2^ at discharge is appropriate based on the AUC analysis and the hazard ratio of multivariate Cox proportional hazards analysis.

Poor ADL tended to worsen long-term mortality, but this finding was not statistically significant in multivariate proportional hazards analysis. Walking ability may be a simple alternative index of ADL and frailty [16–18]. A recent systematic review showed that frailty status, assessed by mobility, disability, and nutritional status, can predict mortality at ≥6 months in older patients undergoing major cardiac surgical procedures [30]. The 6-minute walk test distance [31] and ADL dependence [32,33] are significantly associated with risks of mortality or major adverse cardiovascular and cerebrovascular events after cardiac surgery. In this study, we demonstrated that decreased walking ability may be associated with long-term survival in AKI patients after cardiac surgery. Intervention for frailty may improve long-term survival in these patients, but further study is required.

In this study, BMI <24 kg/m^2^ was associated with poor long-term survival in univariate analysis. Furthermore, when examining the effect of BMI, prognoses in the underweight group (BMI, <18.5 kg/m^2^) were extremely poor compared to those in the overweight group (BMI, 25.0–29.9 kg/m^2^). In our analysis, there was a significant inverse correlation between age and BMI, so confounding might be conceivable. However, it is also possible that the long-term prognosis might be poor when the patients’ underlying cardiovascular disease leads to a state of pre-operative malnutrition. Rahmanian et al. analyzed the impact of BMI on late outcomes in a large series of patients who underwent cardiac surgery. They showed that being underweight (BMI ≤20 kg/m^2^) was an independent predictor of decreased long-term survival [34].

The KDIGO Clinical Practice Guideline for AKI recommends the evaluation of patients 3 months after AKI for resolution, new onset, or worsening of pre-existing CKD [13]. Harel et al. [27] demonstrated that early nephrology follow-up, defined as a nephrology visit within 90 days of discharge, in patients with AKI requiring temporary dialysis was associated with improved long-term survival. However, Siew et al. [35] reported that only 8% of patients hospitalized with an episode of dialysis-requiring AKI see a nephrologist within the first year. Our study indicated that patients with severe AKI requiring CRRT after cardiovascular surgery are at a high risk of chronic dialysis and death, particularly those with eGFR <30 mL/min/1.73 m^2^ at hospital discharge. In the future, it is necessary to construct a system that automatically sends these patients to a nephrologist within 90 days of discharge [36].

This study was limited by a small sample size, and may have been inadequately powered to detect statistically significant differences. Due to the retrospective design, potential confounders, such as indications for initiating CRRT and subsequent management after hospital discharge, were not standardized. Compared with the previous reports, the postoperative CRRT induction rate in our study is high, while in-hospital mortality of those patients is relatively low. Therefore, we believe that improving the quality and quantity of body fluids at an early stage and a strategy of early CRRT initiation will improve the short-term prognosis of AKI. As a result, relatively mild AKI cases may be included, and the differences in the criteria adapted for CRRT initiation may affect long-term prognosis. Furthermore, it is conceivable that including not only cardiac operations but also vascular surgeries in the analysis may have led to the good short-term outcomes. As this was a single-center study, and the majority of participants were elderly, these results may not be generalizable to younger patients or those with different risk profiles. In a large-scale observational study recently published by Ferreiro and Lombardi, age, diabetes and congestive heart failure were found to be associated with survival in 2554 patients with an average age of 67.7 years who developed AKI after cardiac surgery [37]. In addition, in their study, AKI onset was associated with mid-term survival (up to 5 years) but not long-term survival (more than 5 years). Finally, while we indirectly assessed the relationship between comorbidity and long-term prognosis using the CCI, we did not directly analyze the impact of individual factors included in the CCI.

In conclusion, patients with severe AKI requiring CRRT after cardiovascular surgery are at a high risk of chronic maintenance dialysis dependence and mortality. Patients with eGFR <30 mL/min/1.73 m^2^ at discharge in particular should be regularly monitored by a nephrologist due to the risk of chronic dialysis and death. Further study is required to verify that intervention by a nephrologist leads to improved long-term prognosis.
